# Association of Pulmonary Hypertension With End-Stage Renal Disease Among the Obese Population

**DOI:** 10.7759/cureus.9722

**Published:** 2020-08-13

**Authors:** Farah Anum Jameel, Abdul Mannan Junejo, Ayesha Ejaz, Qurat ul ain Khan, Kamran Faisal Bhopal, Ahmad Faraz, Syed Hasan Mustafa Rizvi, Fatima Ahmad, Muhammad Tahir

**Affiliations:** 1 Nephrology, Jinnah Postgraduate Medical Center, Karachi, PAK; 2 Nephrology, Jinnah Postgraduate Medical Centre, Karachi, PAK; 3 Urology, Addenbrooke's Hospital, Cambridge University Hospitals NHS Foundation Trust, Cambridge, GBR; 4 Trauma and Orthopaedics, Leeds Teaching Hospitals NHS Trust, Leeds, GBR; 5 Internal Medicine, Peterborough City Hospital, Peterborough, GBR; 6 Anaesthesia, Punjab Institute of Cardiology, Lahore, PAK; 7 Orthopaedics, Jinnah Postgraduate Medical Center, Karachi, PAK

**Keywords:** obesity, pulmonary hypertension, end stage renal disease, maintenance hemodialysis

## Abstract

Introduction

Pulmonary hypertension (PH) is a known complication that occurs in patients of end-stage renal disease (ESRD) that have an arteriovenous fistula (AVF) for hemodialysis (HD). It is defined as pulmonary artery pressure (PAP) of greater than 30 mmHg on echocardiography. The presence of PH in ESRD is an independent risk factor and decreases the survival likelihood among HD patients. Unexplained PH is frequently seen in ESRD following AVF.

Obesity can lead to various complications, such as sleep apnea, cardiac complications, pulmonary hypertension, and mortality. Data on the prevalence of coexisting PH and obesity are scarce.

Obese patients often have increased albumin excretion rates (AER) that can lead to early renal impairment and an increase in intraglomerular pressure, which may increase the risk of cardiovascular (CV) morbidity and mortality.

Therefore, the study aimed to evaluate and compare the associated PH and obesity separately and collectively among ESRD patients.

Methods

This comparative cross-sectional study was conducted in a tertiary care public sector hospital with the approval of the medical ethics review board committee.

The study enrolled all consecutive patients with ESRD as defined by having an estimated glomerular filtration rate (GFR) of <15 mL/min/1.7 3 m^2^ from April 2017 till March 2019, who presented to our facility. These patients underwent dialysis twice or thrice a week, each session lasting three to four hours approximately. On initial encounter, trans-thoracic echocardiography (TTE) was done by the cardiologist to diagnose pulmonary hypertension.

In addition, body mass index (BMI) was calculated for all patients, and the patients were categorized into underweight, normal, overweight, or obese. All patients underwent post-dialysis TTE at one hour or when patients were at the optimal dry weight. Systolic PAP and ejection fraction were measured, and pulmonary hypertension was defined as a PAP of 30 mmHg or greater on TTE.

ESRD patients that were diagnosed with PH prior to hemodialysis or had primary PH were excluded from the study. Only ESRD patients developing secondary PH after hemodialysis were included in the study.

The chi-square test was used to see the correlation of gender, ambulation status, smoking status, obesity, pulmonary hypertension, body mass index (BMI), and pulmonary hypertension and obesity combined on the final outcome. A p-value of 0.05 was considered significant. Odds ratio (OR) and relative risk (RR) were calculated for pulmonary hypertension and obesity combined, obesity, and pulmonary hypertension in the final outcome.

Results

The study enrolled 204 patients with a mean age of 46.23 (±20.45 SD) having higher female participation of 108 (52.9%), whereas 96 (47.1%) were males. The average weight of the cohort was 66.78 kg (±22.98 SD) with a mean BMI of 29.91 kg/m^2^ (±13.29SD), 52 (25.5%) patients were underweight, 40 (19.6%) had a normal BMI, 29 (14.2%) were overweight, and 83 (40.7%) patients were obese.

Pulmonary hypertension and obesity combined were observed in 48 (23.5%) of the cases and there was a 4.60 relative risk of death among these individuals, with an odds ratio of 13.35 and a p-value of 0.00.

Conclusion

The study shows a strong synergistic effect of pulmonary hypertension and obesity towards the final survival outcome in ESRD patients who are on hemodialysis.

## Introduction

Pulmonary hypertension (PH) is a known complication that occurs in patients with end-stage renal disease (ESRD) who have an arteriovenous fistula (AVF) for hemodialysis (HD). PH is defined as pulmonary artery pressure (PAP) of greater than 30 mmHg on echocardiography [1]. It is a progressive disorder with increased morbidity and mortality [2]. The presence of PH in ESRD is an independent risk factor and decreases the survival likelihood among HD patients [3]. The prevalence of PH in ESRD patients having AVF ranges between 27% and 58% [4]. PH among ESRD patients is a multifactorial process, and echocardiography has a limited ability to define the particular contribution of cardiac output (CO), pulmonary capillary wedge pressure (PCWP), and pulmonary vascular resistance (PVR) to the elevated PAP [5-6]. The postulated mechanism by which PH is developed in ESRD with an AVF is thought to be due to hormonal and metabolic derangements mostly occurring in the endothelium, which leads to an increased pulmonary vascular resistance and increased pulmonary vasoconstriction [7-8]. In addition, AVF itself leads to a high cardiac output, which can be further exaggerated by anemia and fluid overload [9].

Therefore, the World Health Organization (WHO) has categorized PH into five diagnostic groups [10]. Group 1 is pulmonary arterial hypertension (PAH), which can be idiopathic, hereditary, or due to drugs, human immunodeficiency virus (HIV) infection, or being a connective tissue defect; overlapping syndromes - HIV, scleroderma, nephrogenic sclerosing dermatopathy, and end-stage liver disease - are seen in ESRD. Group 2 is pulmonary hypertension secondary to left-sided heart failure, which is often seen in systolic dysfunction, diastolic dysfunction, or valvular heart disease patients; there is a high prevalence of systolic and diastolic heart failure in ESRD patients. Group 3 is pulmonary hypertension due to the disorders of the respiratory system such as chronic pulmonary obstructive, interstitial lung disease, sleep apnea, and obesity; there is a high prevalence of sleep apnea and chronic obstructive pulmonary disease (COPD) in ESRD patients; Group 4 is due to a thromboembolic cause either of the proximal or distal pulmonary vasculatures, mostly seen in patients with pulmonary embolism following AV access thrombectomy, and Group 5 is due to unclear or multifactorial mechanisms seen in myeloproliferative disorders, sarcoidosis, glycogen-storage disease, and chronic kidney disease. Unexplained PH is frequently seen in CKD/ESRD following AVF.

Obesity can lead to various complications, such as sleep apnea, cardiac complications, and pulmonary hypertension, as described by Group 3 of the WHO Classification for PH, and can also result in mortality of individuals [11-12]. Data on the prevalence of coexisting PH and obesity are scarce. However, McQuillan et al. reported that 5% of otherwise healthy individuals with a BMI of greater than 30 kg/m^2^ had moderate or severe pulmonary hypertension on echocardiography [13].

Obesity is a potential risk factor for kidney disease and is associated with abnormal renal parameters [14]. Obese patients often have increased albumin excretion rates (AER) that can lead to early renal impairment and an increase in intraglomerular pressure, which may increase the risk of cardiovascular (CV) morbidity and mortality [15]. Furthermore, dyslipidemia that is associated with obesity leads to progressive chronic kidney disease (CKD) by promoting inflammation and endothelial dysfunction, which also contributes to PH [16].

Therefore, the study aimed to evaluate and compare the associated PH and obesity separately and collectively among ESRD patients.

## Materials and methods

Study demographics

The study was conducted in a tertiary care public sector hospital with the approval of the medical ethics review board committee, and all participants signed informed consent before entering the study.

Study design

This was a comparative cross-sectional study done to evaluate the combined effects of pulmonary hypertension and obesity on the mortality of ESRD patients undergoing hemodialysis.

Study population

The study enrolled all consecutive patients with ESRD as defined by having an estimated GFR of <15 mL/min/1.7 3m2 from April 2017 till March 2019 who presented to our facility. These patients underwent dialysis twice or thrice a week, each session lasting three to four hours approximately.

Study protocol

On initial encounter, trans-thoracic echocardiography (TTE) was done by the cardiologist to diagnose pulmonary hypertension.

Inclusion and Exclusion Criteria

ESRD patients who were diagnosed with PH prior to hemodialysis or had primary PH were excluded from the study. Only ESRD patients developing secondary PH after hemodialysis were included in the study.

In addition, BMI was calculated for all patients and patients who were categorized into underweight - less than 18.5 kg/m^2^, normal weight with a BMI between 18.5 and 24.9 kg/m^2^, overweight if BMI was 25-29.9 kg/m^2^, and obesity was defined as BMI of 30 kg/m^2^ or greater according to the WHO guidelines. All patients underwent post-dialysis TTE at one hour or when patients were at the optimal dry weight. Systolic PAP and ejection fraction were measured and pulmonary hypertension was defined as PAP of 30 mmHg or greater on TTE.

Age, gender, smoking status, etiology of renal failure, ambulation status, weight, duration of treatment, and final outcome (dead or alive) were also recorded. Patients were also categorized as having obesity, developed pulmonary hypertension, both (pulmonary hypertension and obesity), or not.

Final outcome

 The final outcome was mortality.

Statistical analysis

Data were anonymized, and the analysis was conducted on SPSS version 22 (IBM Corp, Armonk, USA). Frequencies and percentages were calculated for ordinal data, whereas for numerical data, mean and standard deviations were obtained from the average values of the age, weight, BMI, and PAP from the first HD session till the study period or the final outcome of death.

The chi-square test was used to see the correlation of gender, ambulation status, smoking status, obesity, pulmonary hypertension, BMI, and pulmonary hypertension and obesity combined on the final outcome. A p-value of 0.05 was considered significant. Odds ratio (OR) was calculated for pulmonary hypertension and obesity combined, obesity, and pulmonary hypertension on the final outcome.

## Results

The study enrolled 204 patients, with a mean age of 46.23 (±20.45 SD) having higher female participation of 108 (52.9%), whereas 96 (47.1%) were males. The average weight of the cohort was 66.78 kg (±22.98 SD) with a mean BMI of 29.91 kg/m^2^ (±13.29SD), 52 (25.5%) patients were underweight, 40 (19.6%) had a normal BMI, 29 (14.2%) were overweight, and 83 (40.7%) patients were obese.

For the cause of dialysis, diabetics were 41 (20.1%), liver failure 33 (16.2%), obstetric sepsis 33 (16.2%), hypertension 32 (15.7%), lupus nephritis 17 (8.3%), heart failure 13 (6.4%), glomerulonephritis 12 (5.9%), lung failure 10 (4.9%), chronic pyelonephritis seven (3.4%), and other cause six (2.9%). Overall, 146 (71.6%) people were alive and 58 (28.4%) died. Table 1 shows the relationship between different variables on the final outcome.

**Table 1:** Cross-tabulation of categorical variables against the final outcome (dead or alive) n/%: number of cases/ percentages; BMI: body mass index; PH: pulmonary hypertension; NS: not significant (p-value greater than 0.05)

Variable	Subcategory	Dead (n/%)	Alive (n/%)	p-value	Chi-square
Gender	Male	26 (44.8)	70 (47.9)	NS	0.16
	Female	32 (55.2)	76 (52.1)		
BMI	Underweight	13 (22.4)	39 (26.7	0.01	17.61
	Normal	6 (10.3)	34 (23.3)		
	Overweight	3 (5.2)	26 (17.8)		
	Obese	36 (62.1)	47 (32.2)		
Obesity	Yes	36 (62.1)	47 (32.2)	0.00	15.35
	No	22 (37.9)	99 (32.2)		
PH	Yes	39 (67.2)	27 (18.5)	0.00	45.07
	No	19 (32.8)	119 (81.5)		
Obesity and PH	Yes	34 (58.6)	14 (9.6)	0.00	55.46
	No	24 (41.4)	132 (90.4)		
Smoker	Yes	17 (29.3)	46 (31.5)	NS	0.09
	No	41 (70.7)	100 (68.5)		
Ambulation status	Independent	6 (10.3)	60 (41.1)	0.00	17.93
	Assisted	52 (89.7)	86 (58.9)		

Furthermore, 66 (32.4%) patients developed pulmonary hypertension during hemodialysis, whereas 138 (67.6%) required assisted ambulation. The mean PAP recorded on TTE was 34.89 (±16.89 SD). Sixty-three (30.9%) of the population were smokers.

Pulmonary hypertension and obesity combined was observed in 48 (23.5%) of the cases and there was a 4.60 relative risk of death among these individuals with an odds ratio of 13.35 and a p-value of 0.00. Table 2 shows the odds ratio along with the correlation of pulmonary hypertension, obesity, and the combined effect of pulmonary hypertension and obesity on the final outcome (mortality).

**Table 2:** Comparing the odds ratio of mortality among patients with pulmonary hypertension and obesity PH: pulmonary hypertension

Variables	Odds ratio	p-value	
PH	9.04	0.00	
			Obesity	3.447	0.00	
PH and obesity	13.35	0.00	

## Discussion

Several isolated studies have been conducted on PH and obesity in ESRD and the effect they have on mortality in ESRD [17-18]. This study explores the collective association of pulmonary hypertension and obesity on the final outcome (mortality) in ESRD on HD.

Pulmonary hypertension (PH) is commonly seen in patients of CKD and ESRD [19-20]. O’Leary et al. attributed that CKD was associated with a 1.4-fold increased risk of having PH. He also stated that mortality significantly increased as the PH status increased. Furthermore, the presence of PH was related to a significant risk of mortality in patients with stage 3 or worse CKD [18,21]. In our study, 19.11% of ESRD patients died having PH with a respiratory rate (RR) of 4.29. The gap in O’Leary’s study was the inclusion of patients having either primary PH or secondary PH. This leaves a potential question: whether there are a cause and effect relation between PH and mortality among CKD patients and fails to answer that PH by itself leads to an increased risk of mortality secondary to right heart failure or it is an independent marker of the severity of the underlying co-morbidities and a predictor of mortality.

In another study, Navaneethan et al., in his multivariate study, reported a 21% prevalence of PH, with a high rate of PH associated with poor kidney function, and PH and high tricuspid regurgitant velocity and PAP were associated with adverse outcomes in CKD. In addition, Navaneethan et al. also established that PH was independently associated with increased risk for death (hazard ratio 1.38) [19].

Our study, on the other hand, only includes cohorts with secondary PH developed after the initiation of HD and tries to fill this void, even though a better model is required to study the cause and effect of PH on the mortality of ESRD patients.

Over the past decade, the prevalence of obesity has increased among adults with ESRD to 10% in the Netherlands [22]. Obesity is an established risk factor for diabetes and hypertension, the top two causes of ESRD in the Netherlands and the United States, and obesity also contributes to cardiovascular disease [23-24]. Snyder et al. examined the association of BMI with time-dependent survival in ESRD and reported the deleterious longer-term effects of higher BMI [25]. This observation was supported by Hoogeveen et al. who reported that increased levels of BMI were associated with a high risk for death in ESRD [26].

In our cohort, 40.6% were obese, out of which 17.64% died. This inference is related to the previous studies conducted to see the association of mortality and obesity in ESRD.

When we studied the combined effect of PH and obesity, we found that 16.6% ESRD patients had died, which is less than the independent effect of PH and obesity but the OR was 13.3 as compared to 9.04 and 3.44 for PH and obesity, respectively. However, we are unable to identify the reason for this but we hypothesize that obesity is pro-inflammatory and leads to endothelial dysregulation, which can ultimately lead to PH and can aggravate cardiac events [27]. Furthermore, it is well-established that both are independently a risk factor for mortality in ESRD patients, and both can independently lead to cardiovascular-related mortality [19,21].

The limitations of this study were that it was a single-center study and the sample size was small as compared to large trials and previous studies. Second, we did not address the obesity paradox in this study and did not include the body fat percentage; This may have different results on the final outcome as per the previous literature dictates [26].

## Conclusions

The study shows a strong synergistic effect of pulmonary hypertension and obesity towards the final survival outcome in ESRD patients who are on hemodialysis. The odds of mortality increase if pulmonary hypertension and obesity co-exist in patients who are on hemodialysis at the end stage of renal failure.
